# Neutrophils and Macrophages: the Main Partners of Phagocyte Cell Systems

**DOI:** 10.3389/fimmu.2012.00174

**Published:** 2012-07-04

**Authors:** Manuel T. Silva, Margarida Correia-Neves

**Affiliations:** ^1^ Instituto de Biologia Molecular e Celular (IBMC), Universidade do Porto,Porto, Portugal; ^2^ Life and Health Sciences Research Institute (ICVS), School of Health Sciences, University of Minho,Braga, Portugal; ^3^ ICVS/3B’s – PT Government Associate Laboratory,Braga/Guimarães, Portugal

**Keywords:** phagocyte systems, microbial infections, neutrophils, monocytes, macrophages, DCs

## Abstract

Biological cellular systems are groups of cells sharing a set of characteristics, mainly key function and origin. Phagocytes are crucial in the host defense against microbial infection. The previously proposed phagocyte cell systems including the most recent and presently prevailing one, the mononuclear phagocyte system (MPS), grouped mononuclear cells but excluded neutrophils, creating an unacceptable situation. As neutrophils are archetypical phagocytes that must be members of comprehensive phagocyte systems, Silva recently proposed the creation of a myeloid phagocyte system (MYPS) that adds neutrophils to the MPS. The phagocytes grouped in the MYPS include the leukocytes neutrophils, inflammatory monocytes, macrophages, and immature myeloid DCs. Here the justifications behind the inclusion of neutrophils in a phagocyte system is expanded and the MYPS are further characterized as a group of dedicated phagocytic cells that function in an interacting and cooperative way in the host defense against microbial infection. Neutrophils and macrophages are considered the main arms of this system.

## INTRODUCTION

When infectious agents pass the peripheral defenses and invade sterile body territories they face innate antimicrobial mechanisms. The pathological result of the presence of a microbe within a host is dependent on the virulence of the pathogen and on the defense competence of the host (Finlay and Falkow, 1989; Casadevall and Pirofski, 2001). When functioning as a pathogen, the infectious agent is living from its pathogenicity, but when the host antimicrobial protective mechanisms dominate there is the efficient protective intervention of several immune and non-immune cells. This intervention is crucially centered on the antimicrobial activities of phagocyte cells, mainly macrophages and neutrophils. Although epithelial cells, fibroblasts, and other cells can phagocytose, in this text the term phagocytes is used for cells whose main function is phagocytosis and that have been classically called professional or dedicated phagocytes, namely neutrophils, inflammatory monocytes, macrophages, and immature dendritic cells (DCs).

Phagocytosis is the process by which eukaryotic cells engulf large particles (>0.5 μm) including prokaryotic and eukaryotic cells. When functioning as a microbe-killing mechanism, phagocytosis triggers rich antimicrobial processes that use a large array of mechanisms involving oxidants like reactive oxygen/nitrogen species (ROS, RNS), granule proteins, and iron-withholding molecules (reviewed in Flannagan et al., 2009). Although phagocytes operate using phagocytic and non-phagocytic mechanisms (Silva, 2010a,b), phagocytosis is the activity that confers their typical character.

## METCHNIKOFF, PHAGOCYTES, AND PHAGOCYTOSIS

While lower eukaryotes use phagocytosis mainly for the acquisition of nutrients, phagocytosis in metazoans is primarily carried out by the specialized professional phagocytes, macrophages, and neutrophils, for a wide range of tasks that include, but are not restricted to, uptake and destruction of invading pathogens (Dale et al., 2008; Cassatella et al., 2009; Silva, 2010a,b).

Although the uptake of particles by cells has been reported before, Metchnikoff, stimulated by Carl Claus, introduced the term “phagocyte,” and the theory of phagocytosis and phagocytic cells is a fundamental contribution by Metchnikoff. His seminal observations behind the phagocytosis theory are extensively discussed in two publications translated to English (Metchnikoff, 1893, 1905) and in biographic texts (see for example Tauber, 2003; Kaufmann, 2008). Metchnikoff understood that phagocytosis represents a central defense mechanism of the host against microbial invaders. The main roots of the theory of phagocytic cells originated from the initial observations made in starfish larva and later in vertebrates. In 1984, working with natural yeast infection in Daphnia, he reported on the antimicrobial activity of phagocytes. In 1887, he described the two types of professional phagocytes in vertebrates, macrophages and neutrophils, the latter initially called “microphages” (Metchnikoff, 1893). Another important Metchnikoff’s contribution to the field of phagocytosis was the understanding that the lamprey’s larvae (ammocoetes) was the lowest biological entity where macrophages and neutrophils coexisted (Metchnikoff, 1905).

The morphological antinomy macrophage/microphage originated by Metchnikoff, based on the differences in morphological size of the two phagocytes (Metchnikoff, 1893), does not have a corresponding parallel with respect to their function. Indeed, when considering the roles of the two phagocytes against microbial infection, Metchnikoff calls neutrophils “principal combatants” and throughout his publications and lectures he stressed that both macrophages and neutrophils were important players: “Phagocytosis is exhibited not only by the macrophages but also, in a high degree, by the microphages which stand out as the defensive cells *par excellence* against microorganisms” (Metchnikoff, 1905).

## NEUTROPHILS: ARCHETYPES OF PHAGOCYTE CELLS

In spite of the importance Metchnikoff attributed to neutrophils as a phagocyte archetype, the post-Metchnikoff evolving trend was to consider macrophages the essential phagocytes minimizing the importance of neutrophils. This macrophage-centric view was reflected in the exclusion of the neutrophil in the initial attribution, in 1967, of the label “professional phagocytes” to macrophages (Rabinovitch, 1967) and in the creation of systems of phagocytic cells that excluded the neutrophil (Aschoff, 1924; Volterra, 1927; van Furth et al., 1972). This cell was considered at the time a terminally differentiated phagocyte with very limited functional capacities. However, the evolution of knowledge about phagocytes progressively led to a new concept of neutrophils as archetypical phagocytic and modulator immune cells, with an origin common to that of the macrophages (Cassatella, 1995, 1999; Rabinovitch, 1995; Akashi et al., 2000; Cassatella et al., 2009; Borregaard, 2010; Silva, 2010b; Costantini and Cassatella, 2011; Mantovani et al., 2011). This new perspective of the neutrophil highlighted novel unexpected capabilities for this essential component of the immune system, which is still under further study nowadays.

Although varying among mammals, the antimicrobial capacity of neutrophils is higher than that of macrophages (Levy, 2004; Segal, 2005). Neutrophils are equipped with a huge assortment of microbicidal mechanisms and use multiple antimicrobial molecules stored in enormous amounts in granules (Borregaard and Cowland, 1997; Segal, 2005). Production of ROS is most prominent in neutrophils as compared with macrophages (Nathan and Shiloh, 2000). Several antimicrobial proteins that are an important part of the neutrophil arsenal are lacking or scarce in the tissue macrophage (Levay and Viljoen, 1995; Lehrer and Ganz, 2002; Selsted and Ouellette, 2005). This is the case of defensins and cathelicidins, the major families of mammalian antimicrobial peptides of neutrophils (Ganz, 2003), and of lactoferrin (Levay and Viljoen, 1995). The bactericidal/permeability-increasing protein (BPI) is also specific of neutrophils (Weiss and Olsson, 1987). Myeloperoxidase (MPO), which is an important enzyme involved in oxidative antimicrobial mechanisms of neutrophils, is present in circulating mammal monocytes but is lost as these mature into macrophages (Klebanoff, 2005), which correlates with decay in antimicrobial activity (Locksley et al., 1987).

## PHAGOCYTE CELL SYSTEMS

Biological cellular systems are groups of cells that share common features, mostly function and origin (Aschoff, 1924; van Furth et al., 1972). Following the pioneer studies by Metchnikoff several systems of phagocytic cells have been created and, strangely, all these were confined to mononuclear cells and excluded neutrophils. One argument used in the proposal of these mononuclear systems was that Metchnikoff considered macrophages as the major phagocytic cells, an interpretation that, as discussed above, is not justified. Aschoff (1924) created the “reticuloendothelial system” (RES), formed by mononuclear cells with presumed phagocytic capacity but neutrophils were left outside for not being considered major phagocytes (Aschoff, 1924). Following progressive criticisms to the previous systems, the “mononuclear phagocyte system” (MPS) was created in 1969 based on a proposal by van Furth and coworkers at a Conference dedicated to Mononuclear Phagocytes. This proposal was formally published (van Furth et al., 1972) and described the MPS as a system of dedicated phagocytic cells with similar morphology, function, origin and kinetics, grouping monocytes/macrophages, and their precursors but again excluding neutrophils. This exclusion was based on the argument that “although polymorphonuclear phagocytes are mononuclear too, they belong to another cell line because of their different origin and divergent kinetic and functional behavior” (van Furth et al., 1972). When the MPS was proposed, knowledge on myelogenesis was limited and the common origin of neutrophils and macrophages was not known, the neutrophils being considered to belong to a cell line separated from that of the MPS members and to be a terminally differentiated phagocytic effector with limited kinetic and functional capacities. 

**FIGURE 1:**
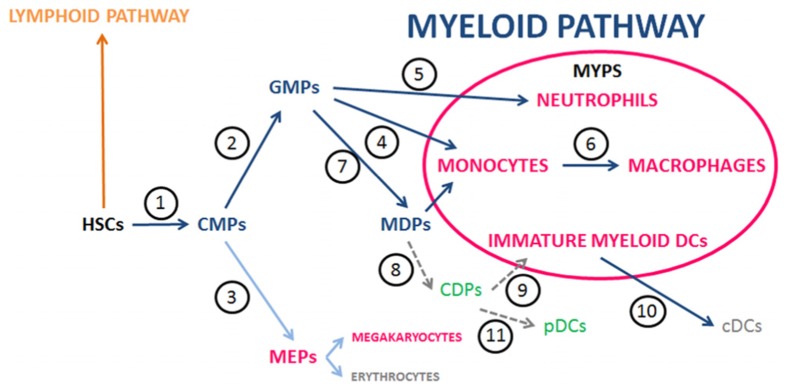
** Main steps and players of myelopoiesis in the context of the MYPS.** Graph depicting one interpretation of the still evolving view of the myelopoiesis pathways (blue arrows) associated with the poiesis of the members of the MYPS in mice and humans. This interpretation takes into consideration recent publications whose references are given below (see also the reviews Friedman, 2002; Iwasaki and Akashi, 2007; Varol et al., 2009; Geissmann et al., 2010b). HSCs give rise to CMPs (1) (Akashi et al., 2000; Manz et al., 2002). CMPs originate GMPs (2) and MEPs (3) (Akashi et al., 2000; Manz et al., 2002). GMPs differentiate into **Monocytes** (4), Eosinophils, Basophils, Mast cells, and **Neutrophils** (5) (Akashi et al., 2000; Iwasaki and Akashi, 2007) and MDPs (7) (Iwasaki and Akashi, 2007). An alternative to the view that MDPs originate from GMPs considered that MDPs would directly derive from CMPs; Fogg et al., 2006). Monocytes mature into **Macrophages** (6) (van Furth et al., 1973; Akashi et al., 2000; Sunderkotter et al., 2004; Fogg et al., 2006). The pathways to myeloid DCs include MDPs [originated from GMPs (7); Iwasaki and Akashi, 2007] that differentiate into CDPs (8) (Onai et al., 2007; Liu et al., 2009) and these give rise to **Immature myeloid DCs** (9) [that originate cDCs (10)], and pDCs (11) (Naik et al., 2006, 2007). The members of the MYPS are highlighted in bold. MYPS, myeloid phagocyte system; HSCs, hematopoietic stem cells; CMPs, common myeloid precursors; GMPs, granulocyte/macrophage precursors; MEPs, megakaryocyte/erythrocyte progenitors; MDPs, macrophage/DC progenitors; CDPs, common DC progenitors; cDCs, conventional (or classic) myeloid DCs; pDCs, plasmacytoid DCs.

Comprehensive phagocyte cell systems must include neutrophils and Silva recently proposed an enlarged system grouping dedicated phagocytic cells including neutrophils (Silva, 2010a). All dedicated phagocytes are of the myeloid lineage (**Figure 1**) as they originate in the “common myeloid progenitors” (CMPs) via the “granulocyte/macrophage lineage-restricted progenitors” (GMPs; Akashi et al., 2000; Iwasaki and Akashi, 2007). Thus, the new system was labeled myeloid phagocyte system (MYPS; Silva, 2010a). Here the justifications for such proposal are extended and the MYPS are further characterized focusing on human and mouse studies. 

## THE MEMBERS OF THE MYPS

The leukocytes of the MYPS share origin, avid phagocytic abilities, and kinetics. Regarding their morphology, the precursor forms of all members of the MYPS are similar except for the mature circulating neutrophil. Mature neutrophils are mononuclear like the other members of the MYPS but the terminal mature form is morphologically differentiated and has a polylobed nucleus. This has been considered as a structural specialization that facilitates the mechanical deformation, allowing neutrophils to “squeeze” through tight spaces when moving to inflammatory/infectious foci.

The list of MYPS members proposed below may have to be altered according to the continuously evolving knowledge in this area. This is particularly relevant for DCs since they share to a great extent the origin and function of other cell members of the MYPS. Of notice, all except neutrophils are antigen-presenting cells. However, since the concept of MYPS, as it stands presently, requires a high phagocytic profile, among the DC cell populations, only immature myeloid DCs were included.

### NEUTROPHILS

All granulocytes are phagocytic but neutrophils (mouse and human key markers: CD66b, LY6-G) are the only exhibiting avid phagocytosis. As already stressed, neutrophils have been acquiring a progressive status of fundamental phagocytic immune cells (Cassatella, 1995, 1999; Rabinovitch, 1995; Cassatella et al., 2009; Borregaard, 2010; Silva, 2010b; Costantini and Cassatella, 2011; Mantovani et al., 2011) with phagocytic abilities superior to those of macrophages (see Section Neutrophils: archetypes of phagocyte cells). Comprehensive reviews on these leukocytes include recent contributions (Nathan, 2006; Nauseef, 2007; Dale et al., 2008; Borregaard, 2010).

### INFLAMMATORY MONOCYTES

Inflammatory monocytes constitute a heterogeneous group of macrophage progenitors and have been divided into two main subsets: a short-lived “inflammatory subset” (key markers; mouse: CD11b, F4/80, Ly6 C; human: CD11b, LY6C) that homes to inflamed tissue, and a “resident subset,” with a longer half-life, that homes to non-inflamed tissues (Geissmann et al., 2003). Inflammatory monocytes are phagocytic and use this capacity as an antimicrobial mechanism (Grage-Griebenow et al., 2001; Sunderkotter et al., 2004; Serbina et al., 2008; Soehnlein et al., 2008c).

### MACROPHAGES

Macrophages (key markers; mouse: CD11b (Mac-1), F4/80; human: CD33) have remarkable phagocytic abilities that largely surpass their contribution to direct antimicrobial host mechanisms of defense. Many recent comprehensive reviews on these phagocytes are available (Hume, 2006; Mosser and Edwards, 2008; Serbina et al., 2008; Pluddemann et al., 2011).

### IMMATURE MYELOID DCs

Dendritic cells (Steinman and Banchereau, 2007) were not included in the MPS based in part on the interpretation that they “are not highly phagocytic” (van Furth et al., 1972). They are a group of primarily antigen presenting and immunomodulatory cells whose distinctiveness has been debated (Hume, 2008; Geissmann et al., 2010a). Mature DCs are not considered dedicated phagocytes and have not been included in the group of professional phagocytes (Rabinovitch, 1995). Mature DCs have a limited capacity for lysosomal degradation of ingested material (Delamarre et al., 2005) and, in contrast to neutrophils and macrophages, are not involved in direct pathogen clearance (Savina and Amigorena, 2007). On the other hand, immature myeloid DCs are phagocytic (Inaba et al., 1993b; Steinman and Swanson, 1995) and have direct effector antimicrobial activities (Banchereau and Steinman, 1998; Banchereau et al., 2000). Thus, they are included as members of the MYPS.

## NEUTROPHILS AS ESSENTIAL MEMBERS OF PHAGOCYTE CELL SYSTEMS

Several arguments justify the inclusion of neutrophils in any comprehensive phagocyte cell system, as follows.

1. Neutrophils are archetypical phagocytes: As referred before, the exclusion of neutrophils from the MPS was based on the interpretation that their origin and kinetic and functional behavior are different from those of monocytes/macrophages, interpretation that, with the advances in knowledge, has been proved incorrect.
2. Neutrophils, monocytes/macrophages and immature myeloid DCs have a common origin: The initial view that neutrophils and macrophages arise from a common late bone marrow precursor (Metcalf, 1989; Inaba et al., 1993a) has been confirmed by results showing that these phagocytes originate from hematopoietic stem cells (HSC) which differentiate through common pathways that also lead to immature myeloid DCs (Akashi et al., 2000; Iwasaki and Akashi, 2007; **Figure 1**).
3. Macrophages and neutrophils share important features: Important features shared by macrophages and neutrophils with respect to their common origin include: (i) avid phagocytic capabilities (Dale et al., 2008); (ii) presence of common surface markers like chemokine receptors (Silva, 2010a) and receptors for Igs and complement (Dale et al., 2008), and common patterns of cytokine and chemokine secretion (Silva, 2010a); (iii) common expression of pattern recognition receptors (PRR) (Janeway, 1989); (iv) cooperative participation in the orchestration of adaptive immune responses (Silva, 2010b); (v) scavenger capacity [while macrophages are the main scavenger phagocyte (Parnaik et al., 2000), neutrophils may function as a backup system when the scavenging capacity of macrophages is overwhelmed in situations of hyper-inflammation (Rydell-Tormanen et al., 2006)]; (vi) similarity on the kinetic behavior under inflammatory/infectious conditions (Silva, 2010a). Also to consider are reports on the possible conversion of neutrophils into macrophages (Araki et al., 2004; Sasmono et al., 2007). Additionally, two functional criteria that were taken into consideration to select cells to be grouped in the MPS, namely pinocytosis and the ability to attach firmly to a glass surface (van Furth et al., 1972), are now known to be exhibited by neutrophils as well (Hoffstein et al., 1981; Davis et al., 1986).
4. Macrophage/neutrophil functional partnership: The cooperation between the members of a cell system enhances its functional efficiency (Seeley, 2002; Shou et al., 2007). Data discussed above and elsewhere (Silva, 2010a,b, 2011) indicate that the MYPS is an assembly of dedicated phagocytic cells that function in an interacting and cooperative way.

While sharing several functions, macrophages and neutrophils are specialized cells with functional and function-related morphological distinctive features. These features are complementary and provide varied levels of antimicrobial capacities and cytotoxicity, and tissue-specific localization and lifespan (Silva, 2010a). These distinctive features explain why macrophages and neutrophils are not able to replace each other as central players of antimicrobial immunity, as indicated by the pathology associated with some human and murine phagocyte deficiencies (Dale and Liles, 2002).

The combination of shared and complementary features of macrophages and neutrophils promotes their cooperative participation as effectors and modulators in immunity against infection (Silva, 2010a,b). This cooperation is clearly illustrated by the ability of macrophages, in their process of killing intracellular bacteria, to take up proteins and peptides (e.g., human neutrophil peptide 1) produced and released by neutrophils. In addition, macrophages are also able to engulf apoptotic neutrophils and make use of the antimicrobial molecules present in their granules. Based on these results (Silva et al., 1989; Sharma et al., 2000; Tan et al., 2006; Sawant et al., 2010), in 2009 Silva proposed the concept of macrophage/neutrophil partnership in the host response against infection (Silva, 2010a), a concept extensively reviewed in Silva (2011).

The framework of the concept of macrophage/neutrophil partnership (Silva, 2010a,b, 2011), which is a central facet of the MYPS system, includes several cooperative macrophage/neutrophil activities. (i) At the initiation of the infectious inflammation, complex networks of cytokines and chemokines originate through interaction of monocytes/macrophages and neutrophils at the infectious/inflammatory foci (Silva, 2010a; Soehnlein and Lindbom, 2010). (ii) Amplification of the limited antimicrobial capacity of macrophages by the acquisition of neutrophil potent microbicidal molecules without transit as extracellular damage associated molecular pattern molecules (DAMPs), thus reducing the harm due to excessive inflammation (Silva et al., 1989; Silva, 2010a). In situations where macrophages handle diverse intramacrophage microbes in different ways, antimicrobial components of neutrophil granules, acquired by macrophages through uptake of neutrophils or neutrophil granules, are mobilized to the diverse types of microbe-containing vacuoles (Silva, 2011). At these locations the acquired molecules may exert their antimicrobial role through a direct activity against the intramacrophage pathogens (Sharma et al., 2000; Tan et al., 2006) or through interaction with the endogenous macrophage antimicrobial mechanisms (Lincoln et al., 1995; Marodi et al., 1998). (iii) A non-phagocytic facet of the interactive cooperation macrophage/neutrophil at the effector level is the reciprocal activation of monocytes/macrophages and neutrophils. Monocytes/macrophages can be activated directly by neutrophil products, including released granule molecules, with boosting of their phagocytic and antimicrobial capacities (Lima and Kierszenbaum, 1985; Ichinose et al., 1996; Zughaier et al., 2005; Soehnlein et al., 2008a,b,c) and neutrophils are activated by macrophage-secreted cytokines and chemokines and acquire an inflammatory effector phenotype (reviewed in Silva, 2010a). (iv) Interaction and cooperation results in the resolution of the infectious inflammation upon control of the infectious process, as well (Silva, 2010a; Soehnlein and Lindbom, 2010). A similar concept was proposed after the initial paper by Silva (Soehnlein and Lindbom, 2010).

This cooperative partnership represents a factor for increased efficiency of the MYPS. As already noted by Metchnikoff (1905), the presence of two professional phagocytes is exclusive of the immune system of vertebrates. The cooperation macrophage/neutrophil within the competences of the MYPS, allows an immune attack strategy against microbial infections based on two complementary phagocytes that safely take advantage of powerful neutrophil microbicidal factors that are transferred to the infected macrophage. This is a safe way of macrophages to make use of powerful but dangerous microbicidal molecules avoiding the problems of permanently carrying these cytotoxic factors. This strategy is a target of key virulence mechanisms of successful pathogens.

## CONCLUSION

Based on the principle that phagocyte cell systems must include all dedicated phagocytic cells, the creation of the MYPS (Silva, 2010a) was proposed, changing the unacceptable prevailing situation where the only phagocyte cell system in use (MPS) excludes neutrophils. The members of this system have common origin and share avid phagocytic abilities. Besides individual activities of the members of a cellular system, cooperation between them enhances the system’s functional efficiency. Macrophage/neutrophil partnership, important in phagocytic, immunomodulatory, and inflammation pro-resolving activities, is particularly relevant in the operation of the MYPS (Silva, 2010a). Thus, neutrophils and macrophages are the main arms of this system.

In conclusion, the MYPS is a system of dedicated phagocytic cells that groups neutrophils, inflammatory monocytes, macrophages, and immature myeloid DCs; these functions in an interacting and cooperative way in the host defense against microbial infection.

## Conflict of Interest Statement

The authors declare that the research was conducted in the absence of any commercial or financial relationships that could be construed as a potential conflict of interest.
